# Risk of Gastrointestinal Cancers among Patients with Appendectomy: A Large-Scale Swedish Register-Based Cohort Study during 1970-2009

**DOI:** 10.1371/journal.pone.0151262

**Published:** 2016-03-09

**Authors:** Huan Song, Christian C. Abnet, Åke Andrén-Sandberg, Anil K. Chaturvedi, Weimin Ye

**Affiliations:** 1 Department of Medical Epidemiology and Biostatistics, Karolinska Institutet, Stockholm, Sweden; 2 Division of Cancer Epidemiology and Genetics, National Cancer Institute, Bethesda, Maryland, United States of America; 3 Department of Digestive Diseases, Karolinska University Hospital, Huddinge, Stockholm, Sweden; Institute of Psychiatry, UNITED KINGDOM

## Abstract

**Background:**

Removal of the appendix might induce physiological changes in the gastrointestinal tract, and subsequently play a role in carcinogenesis. Therefore, we conducted a nationwide register-based cohort study in Sweden to investigate whether appendectomy is associated with altered risks of gastrointestinal cancers.

**Methods:**

A population-based cohort study was conducted using the Swedish national registries, including 480,382 eligible patients followed during the period of 1970–2009 for the occurrence of site-specific gastrointestinal cancer (esophageal/gastric/colon/rectal cancer). Outcome and censoring information was collected by linkage to health and demography registers. We examined the incidence of appendectomy in Sweden using data from 1987–2009. We also calculated standardized incidence ratios (SIRs) with 95% confidence intervals (CIs) to estimate the relative gastrointestinal cancer risk through comparison to the general population.

**Results:**

We noted an overall decrease in the age-standardized incidence of appendectomy among the entire Swedish population from 189.3 to 105.6 per 100,000 individuals between 1987 and 2009. Grouped by different discharge diagnosis, acute appendicitis, incidental appendectomy, and entirely negative appendectomy continuously decreased over the study period, while the perforation ratio (18%–23%) stayed relatively constant. Compared to the general population, no excess cancer risk was observed for gastrointestinal cancers under study with the exception of a marginally elevated risk for esophageal adenocarcinoma (SIR 1.32, 95% CI 1.09–1.58).

**Conclusions:**

In Sweden, the incidence of appendectomy and acute appendicitis has decreased during 1987–2009. No excess gastrointestinal cancer risks were observed among these appendectomized patients, with the possible exception of esophageal adenocarcinoma.

## Introduction

Appendectomy is a common procedure, which is usually performed after a clinical suspicion of acute appendicitis. In addition, it can also be conducted incidentally to other operations—that is, the appendix is removed without evidence of disease, as a preventive measure when a subject is undergoing another operation. Incidental appendectomy most commonly occurs during hysterectomy or salpingectomy, and therefore is more prevalent in females [1, 2]. Although few studies have documented this in detail, current data show a slow, long-term decline in incidence, at least in Western countries [3, 4, 5].

The main reasons for the regular use of this operation include: 1) appendicitis is a potential deadly disease; 2) historically, the vermiform appendix was defined as a vestigial organ; and 3) the loss of the appendix is thought to have few, if any, long-term effects. However, recent data indicate that the appendix might serve as a reservoir for the colonic microbiome, and therefore the excision of the appendix may affect the components of gastrointestinal microbiome, which ultimately may play a role in the development of gastrointestinal cancers [6, 7]. Although inconsistent, a few epidemiological studies have reported altered risks of cancer, especially stomach cancer, among patients with an appendectomy history [8, 9]. Moreover, a recent trial suggested that uncomplicated acute appendicitis could be treated with antibiotics rather than appendectomy, highlighting the importance of assessing the sequelae of appendectomy [10].

We revisited the hypothesis that individuals who received appendectomy would experience an increased risk of gastrointestinal cancers, possibly due to the disruption of the normal colonic microbiome, by using our nationwide register-based appendectomy cohort. In addition, we also aimed to examine the secular trend in the incidence of appendectomy among the entire Swedish population.

## Methods

### Databases

Based on the National Patient Registry of Sweden, a cohort consisting of patients born prior to 2003 who received appendectomy during January 1, 1970 to December 31, 2009 was established. The Swedish surgery codes for open or laparoscopic appendectomy procedure were 4510, 4511, 4517, 0058 during the time period 1970–1996, and JEA00, JEA01, JEA10 for 1997 and onwards. The National Patient Registry database was created initially in 1964/1965, and became nationwide since 1987, providing data including national registration number, which is a unique identifier for each individual, personal information (e.g. age, sex, county), dates of admission and discharge, surgical procedure codes, and medical diagnoses. External reviews demonstrated that, more than 50% of all surgeries were reported to the National Patient Registry since 1976, and the complete coverage (100%) was obtained in 1987 [11].

The dataset was then linked to the Swedish Cancer Registry, where all incident gastrointestinal cancer cases (Swedish International Classification of Diseases (ICD) (version 7) = 150 for esophageal cancer, 151 for gastric cancer, 1530-1533/ 1538/1539 for colon cancer (except appendix cancer), and 1540 for rectal cancer) [12] were ascertained. Esophageal cancer was sub-grouped as esophageal adenocarcinoma and esophageal squamous-cell carcinoma, according to the histopathological diagnoses (patho-anatomic diagnosis, PAD, ‘096’ for esophageal adenocarcinoma and ‘146’ for esophageal squamous-cell carcinoma). Gastric cancer coded as ICD-7 1511 was sub-classified as cardia cancer, while the others were counted as non-cardia gastric cancer indicating that the malignancy originated from any other subsite than the cardia. The coding for cardia cancer was introduced in 1969, and widely applied since 1970 [13]. Colon cancer was sub-divided into right-sided colon cancer (ICD7 1530–1531) and left-sided colon cancer (ICD7 1532–1533). The Swedish Cancer Register was established in 1958, with a completeness rate of 98% for gastrointestinal cancers [14]. Cross linkage with the Death Register and Emigration Register provided the necessary information for censoring follow-up.

### Study design

We identified a total of 501,160 patients who underwent appendectomy in Sweden during 1970–2009. After excluding 20,778 patients with conflicting information (having emigration/death date recorded before the appendectomy date), the final cohort consisted of 480,382 eligible subjects. In each sub-cohort which was established specifically for evaluating certain gastrointestinal cancer risk (esophageal/ gastric/colon/ rectum cancer), we further excluded the patients who received a diagnosis of such cancer before the hospitalization for appendectomy. Follow-up for each participant was then started from the date of appendectomy, until certain cancer occurrence, migration out of Sweden, death, or the end of follow-up (December 31, 2009), whichever occurred first.

Moreover, since non-specific symptoms (e.g. abdominal pain) could be present due to an as-yet-undetected cancer, which may consequently increase the likelihood of having appendectomy, selection bias should be taken into consideration for assessing the post-appendectomy cancer risks. Therefore, for all further analyses, the first year of follow-up, as well as the corresponding events happening during that period, was disregarded. This study was approval by the Regional Ethical Review Board in Stockholm. All the individual records were anonymized and de-identified prior to analysis.

#### Appendectomy subgroups

According to the discharge diagnosis, we categorized the appendectomy patients into different subgroups: 1) *perforated or abscessed appendicitis* group involved all patients with the discharge diagnoses coded as (ICD-8) 54000–54003, 54302, or (ICD-9) 540A, 540B or (ICD-10) K35.0, K35.1, K38.3 in their medical records; 2) *acute non-perforated appendicitis* group consisted of patients having diagnosis codes of (ICD-8) 54090–54208, or (ICD-9) 540X, 541, 542, or (ICD-10) K35.9, K36, K37); 3) *entirely negative appendectomy* group with patients who ended up with a diagnosis of mesenterial adenititis or unspecific abdominal pain, and without any other surgical procedure appearing in the same hospitalization record; and 4) *incidental appendectomy* group was defined as patients without appendicitis diagnosis but having additional surgical procedure(s) performed at the same time as the appendectomy.

#### Statistical methods

For describing the temporal trends in the whole Sweden, we focused on the appendectomy patients recorded after 1986 (n = 269,185). We used Sweden resident population estimates for the years 1987–2009 to calculate annual incidence rates of appendectomy, standardized by world standard population (Segi 1960) and presented as appendectomy cases per 100,000 individuals per year. Trends in incidence during the period were analyzed by Poisson regression. We also studied the secular trends among different subgroups (by sex, age group, or underlying diagnoses) separately.

To estimate relative risks of each gastrointestinal cancer, we calculated standardized incidence ratios (SIRs) using the general population as reference (1970–2009). The expected numbers were estimated by multiplying the calendar period-, age-(5 year intervals) and sex-specific follow-up time in the cohort with the corresponding incidence rates derived from the entire Swedish population. We calculated 95% confidence intervals (CIs) under the assumption that the observed number of cases followed a Poisson distribution. Further analyses were stratified by underlying diagnoses, sex, age group (0–19 years, 20–39 years, 40–59 years, 60 years and above), and the duration of follow-up (1–4, 5–9, 10–14, 15–24 and 25+). We conducted trend tests by including the age, follow-up years as continuous variables in Poisson regression models, using the expected number of cases as offset. The fit of the regression model was checked using Pearson’s Chi-square statistic. All analyses were repeated using completely nationwide data only (1987–2009) as sensitivity analyses.

A *P* value less than 0.05 was considered to be statistically significant. All statistical analyses were performed using SAS 9.4 software (Cary, NC).

## Results

### Incidence of appendectomy in Sweden, during 1987–2009

**Fig 1:**
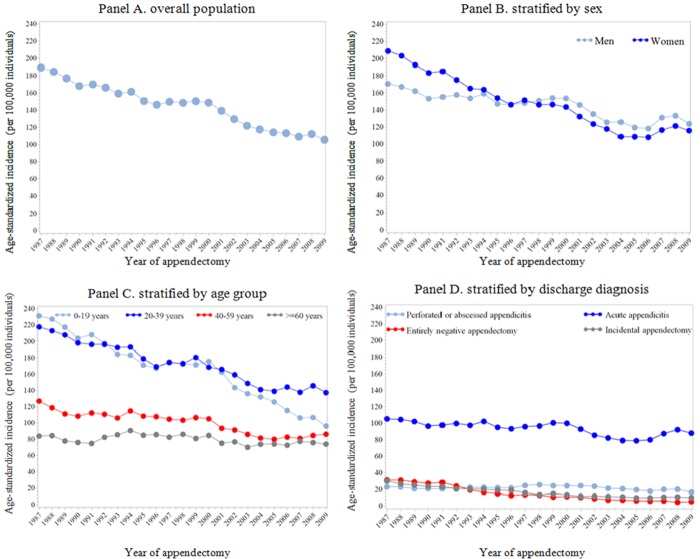
The age-standardized incidence of appendectomy in Sweden, during 1987–2009 (n = 269,185). Panel A. the age-standardized incidence of appendectomy over years among overall population; Panel B. the age-standardized incidence of appendectomy over years, stratified by sex; Panel C. the age-standardized incidence of appendectomy over years, stratified by age group; Panel D. the age-standardized incidence of appendectomy over years, stratified by discharge diagnosis.

**Fig 2:**
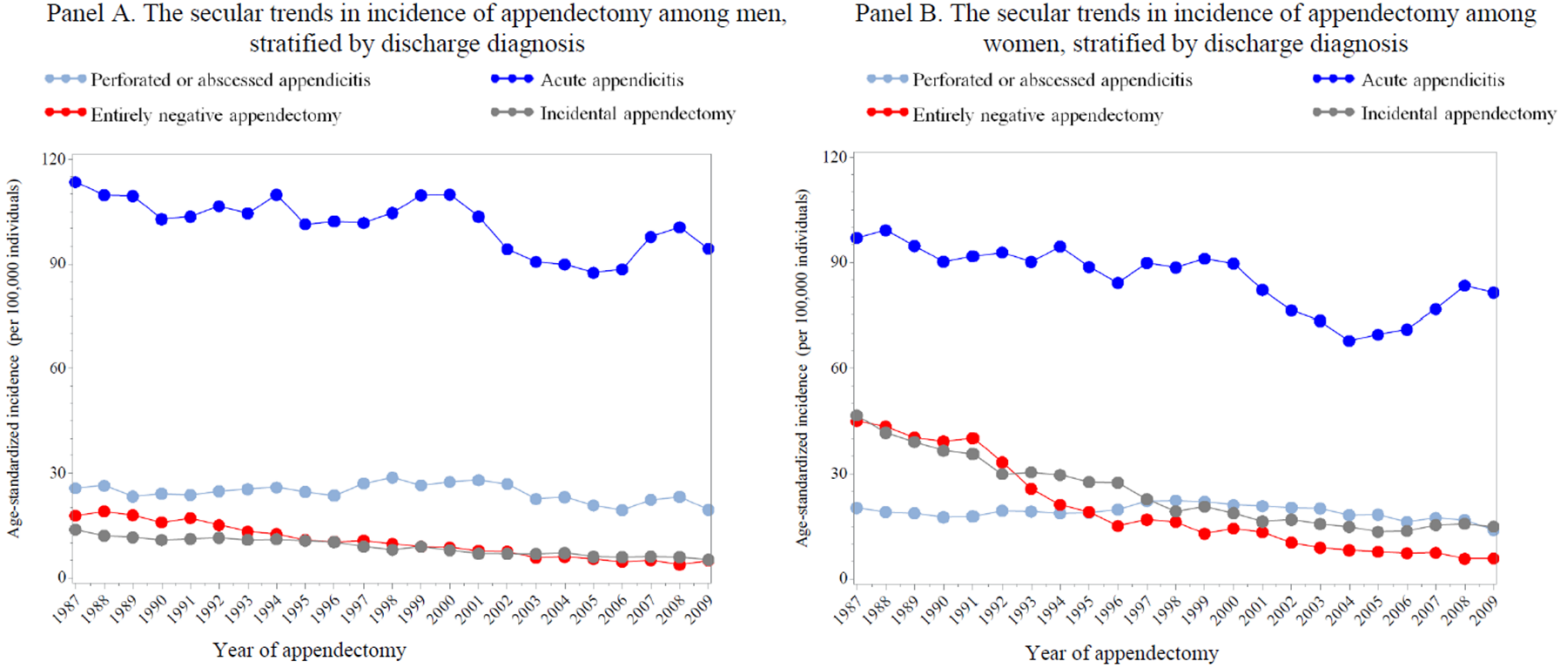
The secular trends in age-standardized incidence of appendectomy among men and women, stratified by discharge diagnosis. Panel A. the secular trends in age-standardized incidence of appendectomy among men, stratified by discharge diagnosis; Panel B. the secular trends in age-standardized incidence of appendectomy among women, stratified by discharge diagnosis.

In total, 269,185 patients who underwent appendectomy during 1987–2009 were identified. Fig 1 illustrates the annual age-standardized incidence of appendectomy among the entire Swedish population over a 22-year span. An overall decreasing trend was noted (Fig 1, Panel A, from 189.3 to 105.6per 100,000 individuals between 1987 and 2009) −− the annual rate of decrease in age-standardized incidence was 2.5% (95% Cl 2.0–3.0%). Between 1987 and 1996, the age-standardized incidence rates of appendectomy for women were higher than those for men, while the opposite pattern was obtained for years after 1997 (Fig 1, Panel B). We therefore observed a more apparently decreasing trend among women, relative to men (*P* for difference < 0.001). The highest age-standardized incidence rate of appendectomy, together with the sharpest decline (from 236.2 to 96.4 per 100,000 between 1987 and 2009, corresponding to an annual decreasing rate of 3.5%; 95% CI 3.0–3.9%<), was observed among patients aged 0–19 years; but for individuals above 60 years old, non-significantly decreasing trends were observed (Fig 1, Panel C). Further stratified by sex, similar age group-specific temporal trends were observed. Grouped by different discharge diagnoses (Fig 1, Panel D), acute appendicitis, incidental appendectomy, and entirely negative appendectomy significantly decreased over the study period, while appendectomies attributed to perforated appendicitis remained unchanged. Notably, despite similar changing patterns regarding the incidence of acute and perforated appendicitis in both sex groups, for appendectomies due to other than appendicitis diagnosis, the annual reduction rates seemed to be higher among women, relative to men (*P* for difference = 0.08 for entirely negative appendectomy, and = 0.10 for incidental appendectomy) (Fig 2).

### Gastrointestinal cancer risks among appendectomy patients

For cancer-risk assessment, during 1970–2009, 480,382 appendectomy patients were identified, with a mean age of 32 years (Table 1). More women were included than men (men: women = 1:1.2) in the whole cohort, which was probably driven by the obvious female-dominance in the other diagnoses subcohort. In the appendicitis cohort, however, 55% subjects were male. Regarding the underlying discharge diagnoses, acute (non-perforated) appendicitis was the most common reason for appendectomy. The entirely negative appendectomy was more frequent among young women (0–29 years), whereas incidental appendectomy tended to prevail among older females (above 40 years old).

**Table 1:** Description of appendectomies in Sweden during 1970–2009, grouped by underlying diagnoses.

	All appendectomy cohort	Appendicitis subcohort	Other diagnoses subcohort
**Number**	480,382	327,496	152,886
**Age, mean± SD (years)**	32.5±19.6	30.2±18.7	37.4±20.6
**Age group, n (%)**			
0–19 years	157,024(32.7)	120,807 (36.9)	36,217 (23.7)
20–39 years	169823(35.3)	120,488 (36.8)	49,335 (32.3)
40–59 years	95527(19.9)	53,841 (16.4)	41,686 (27.3)
60 years and above	58008(12.1)	32,360 (9.9)	25,648 (16.7)
**Sex, n (%)**			
Men	217,710 (45.3)	180,172 (55.0)	37,538 (24.6)
Women	262,672 (54.7)	147,324 (45.0)	115,348 (75.4)
**Calendar year of appendectomy, n (%)**			
1970–1979	113,499 (23.6)	62,270 (19.0)	51229 (33.5)
1980–1989	139,977 (29.1)	88,966 (27.2)	51011 (33.4)
1990–1999	124,677(26.0)	92,167 (28.1)	32510 (21.3)
2000–2009	102,229(21.3)	84,093 (25.7)	18136 (11.8)
**Duration of follow-up**			
**mean± SD (years)**	18.6± 10.9	17.5± 10.6	20.8±11.2
**n (%)**			
0–4 years	66,417 (13.8)	46,989 (14.4)	19,428 (12.7)
5–14 years	127,550 (26.6)	99,038 (30.2)	28,512 (18.6)
15–24 years	134,484 (28.0)	92,666 (28.3)	41,818 (27.4)
> = 25 years	151,931 (31.6)	88,803 (26.1)	63,128 (41.3)

In Table 2, SIRs together with their 95% CIs are presented for each type of gastrointestinal cancers in all appendectomized patients, as well as stratified by discharge diagnoses (appendicitis/ other diagnoses), after excluding the first year of follow-up. We observed no association for cancers of the stomach, colon, or rectum. We found divergent results for esophageal cancers by histopathological type. Appendectomy was associated with an increased risk for esophageal adenocarcinoma (SIR 1.32, 95% CI 1.09–1.58), but a reduced risk for esophageal squamous-cell carcinoma (SIR 0.79, 95% CI 0.65–0.95). Subgroup analysis indicated this reduction to be limited to the stratum of patients with appendicitis diagnosis (SIR 0.71, 95% CI 0.54–0.91), and males (SIR 0.74, 95% CI 0.57–0.96). A sensitivity analysis restricted to complete nationwide data during 1987–2009 revealed similar results as in the primary analysis (with similar point estimates), albeit with wider CIs (S1 Table).

**Table 2:** Standardized incidence ratios (SIRs) and 95% confidence intervals (CIs) for gastrointestinal cancers in appendectomy patients with different discharge diagnoses.

	All appendectomy cohort		Appendicitis		Other diagnoses	
	Observed cases*	SIR (95% CI)^§^	Observed cases*	SIR (95% CI)^§^	Observed cases*	SIR (95% CI)^§^
**Esophageal cancer, all**	255	1.00 (0.88–1.13)	153	0.93 (0.79–1.09)	102	1.14 (0.93–1.38)
Esophageal adenocarcinoma	118	1.32 (1.09–1.58)	79	1.25 (0.99–1.55)	39	1.50 (1.07–2.05)
Esophageal squamous-cell carcinoma	114	0.79 (0.65–0.95)	62	0.71 (0.54–0.91)	52	0.92 (0.69–1.21)
**Gastric cancer, all**	831	1.00 (0.93–1.07)	496	1.01 (0.92–1.10)	335	0.98 (0.87–1.09)
Non-cardia gastric cancer	685	0.99 (0.92–1.07)	393	0.99 (0.90–1.10)	292	0.99 (0.88–1.11)
Cardia cancer	146	1.06 (0.89–1.25)	103	1.11 (0.91–1.35)	43	0.95 (0.69–1.28)
**Colon cancer, all**	2473	1.03 (0.99–1.07)	1373	1.00 (0.95–1.06)	1100	0.98 (0.92–1.04)
Right-sided colon cancer	1282	1.04 (0.99–1.10	693	1.05 (0.97–1.13)	589	1.04 (0.95–1.12)
Left-sided colon cancer	860	0.96 (0.90–1.03)	498	0.99 (0.91–1.08)	362	0.92 (0.83–1.02)
**Rectal cancer**	1280	0.98 (0.93–1.04)	712	0.94 (0.87–1.01)	568	0.94 (0.87–1.01)

*The first year of observation and corresponding events were excluded.

^§^ Observed to expected number of cancer cases, based on age- (5-year strata), calendar year- (5-year strata) and sex-specific incidence data in the total Swedish population. Ninety-five percent CIs of SIRs were calculated by assuming that observed cancer occurrence followed a Poisson distribution.

Stratified by time since operation (Table 3), appendectomy was associated with an increased cardia cancer risk (SIR 1.63, 95% CI 1.12–2.31) in the first 4 years of follow-up. However, this excess quickly disappeared after the initial observation period. We also observed elevated risks for esophageal adenocarcinoma (SIR 1.48, 95% CI 1.06–2.02) and right-sided colon cancer (SIR 1.12, 95% CI 1.05–1.20) between 5–14 years after appendectomy. Whereas for long-term observation (more than 15 years since appendectomy), no excess risk was noted for all of studied gastrointestinal cancers except esophageal adenocarcinoma (≥ 25 years, SIR 1.50, 95% CI 1.06–2.07).

**Table 3:** Standardized incidence ratios (SIRs) and 95% confidence intervals (CIs) for gastrointestinal cancers in appendectomy patients, stratified by duration of follow-up.

	1–4 years		5–14 years		15–24 years		≥25 years		Trend test
	Observed cases*	SIR (95% CI)^§^	Observed cases*	SIR (95% CI)^§^	Observed cases*	SIR (95% CI)^§^	Observed cases*	SIR (95% CI)^§^	*P* value
**Esophageal cancer, all**	37	1.00 (0.70–1.38)	80	0.92 (0.73–1.15)	73	0.97 (0.76–1.22)	65	1.17 (0.90–1.49)	0.30
Esophageal adenocarcinoma	12	1.15 (0.59–2.01)	40	1.48 (1.06–2.02)	29	1.06 (0.71–1.52)	37	1.50 (1.06–2.07)	0.73
Esophageal squamous-cell carcinoma	18	0.79 (0.47–1.25)	36	0.70 (0.49–0.96)	38	0.90 (0.64–1.24)	22	0.80 (0.50–1.22)	0.60
**Gastric cancer, all**	171	1.11 (0.95–1.29)	309	0.97 (0.87–1.09)	214	0.95 (0.83–1.09)	137	1.00 (0.84–1.18)	0.31
Non-cardia gastric cancer	139	1.04 (0.87–1.23)	265	0.99 (0.87–1.11)	176	0.96 (0.83–1.12)	105	0.99 (0.81–1.19)	0.62
Cardia cancer	32	1.63 (1.12–2.31)	44	0.93 (0.67–1.24)	38	0.91 (0.65–1.26)	32	1.10 (0.75–1.55)	0.20
**Colon cancer, all**	342	1.05 (0.94–1.17)	886	1.12 (1.05–1.20)	711	0.98 (0.91–1.06)	534	0.95 (0.87–1.03)	0.01
Right-sided colon cancer	177	1.11 (0.95–1.28)	451	1.15 (1.05–1.26)	358	0.98 (0.88–1.08)	296	0.96 (0.85–1.07)	0.01
Left-sided colon cancer	108	0.88 (0.73–1.07)	301	1.02 (0.91–1.15)	257	0.97 (0.85–1.09)	194	0.92 (0.79–1.06)	0.77
**Rectal cancer**	186	1.02 (0.88–1.18)	442	1.00 (0.91–1.10)	369	0.94 (0.85–1.04)	283	0.99 (0.88–1.11)	0.56

*The first year of observation and corresponding events were excluded.

^§^ Observed to expected number of cancer cases, based on age- (5-year strata), calendar year- (5-year strata) and sex-specific incidence data in the total Swedish population. Ninety-fiver percent CIs of SIRs were calculated by assuming that observed cancer occurrence followed a Poisson distribution.

To evaluate the possibility of confounding due to differential distributions between the appendectomy cohort and the general population with regard to addictive substance (including tobacco and alcohol), which are thought to be important risk factors for some gastrointestinal cancers [15, 16], we also calculated SIRs for tobacco- (lung cancer) and alcohol-related (liver cancer) cancers [15, 17]. The relative risks of lung cancer among the appendectomized patients was almost equal to unity (SIR 1.03, 95% CI 0.98–1.09), whereas the corresponding figure for liver cancer risk showed a 16% reduction (SIR 0.84, 95% CI 0.70–0.98). This reduction was mainly confined to subgroups who were men (SIR 0.72, 95%CI 0.57–0.89), or with appendicitis diagnosis (SIR 0.82, 95% CI 0.68–0.98).

## Discussion

Using a large, nationwide, register-based cohort study with a long follow-up duration (mean follow-up of 18.6 years), we confirmed that in Sweden, the incidence of appendectomy has decreased during last few decades. In addition, compared to the general Swedish population, a slightly increased risk of esophageal adenocarcinoma, contrasted by a reduced esophageal squamous-cell carcinoma risk, was observed among the appendectomized patients; whereas no differences were noted for gastric cancer, colon cancer, and rectal cancer.

For decades, the decreased incidence of appendectomy has been documented, both in Sweden [5] and other Western countries [3, 4]. This decline is most pronounced in younger age groups, and usually accompanied with a falling incidence of appendicitis. Consistent with prior reports [3, 4], both acute (non-perforated) and perforated appendicitis illustrated a male predominance. However, unlike recent US studies [18, 19] which reported an increased incidence of appendicitis, as well as a decreased odds of perforation, our data indicated that the incidence of appendectomy due to acute appendicitis kept decreasing, and the perforation ratio, nevertheless, stayed almost constant over years (about 18%-25%) among operated appendicitis patients. One possible explanation for the inconsistency could be that choice for surgery for acute appendicitis became less frequent in Sweden than before. Our data, however, did not support this statement since a relatively constant operation rate among appendicitis patients was observed during the study period. We noted a reduction in the incidence of entirely negative appendectomy. The detected pattern suggests an improved diagnostic accuracy for non-perforated appendicitis, which may be attributed to more and more frequent use of ultrasonography and computed tomography scanning. The perforation ratio, however, remained unchanged since it’s probably more related to timely surgical intervention, rather than accurate diagnosis [3, 20]. In line with the notion that women with suspected appendicitis benefit the most from pre-operative imaging tests [21, 22], our results suggested a steeper decline of negative appendectomy among females relative to males—the entirely negative appendectomy used to be 3 times more common among women than men; but the sex difference has gradually disappeared, resulting in a low incidence rate of 5 per 100,000 individuals in 2009 for both sexes. In addition, incidental appendectomy was widely performed among old females in the earliest study period because the prevailing view at that time was that it could eliminate the future risk of appendicitis in these elderly women which was associated with a considerable possibility of perforation and death, without adding any additional morbidity [23]. Moreover, gynecologists have always had difficulties with differentiating appendicitis from diseases of the right ovarium and salpinx before the era of ultrasonography which made it tempting to remove the appendix during gynecological surgeries. However, such prophylactic removals gave rise to controversy owning to conflicting results from subsequent clinical studies [24].

Few studies have assessed the association between appendectomy and the risk of subsequent gastrointestinal cancers, and the results are inconsistent. A study using Swedish Patient and Cancer registers (1965–1993) with such a focus [8] found a significantly increased risk of stomach cancer but a significantly reduced risk of colon cancer after appendectomy. However, that study was limited to subjects who had their appendix removed before 20 years of age. With the limited follow-up duration (the oldest patient was younger than 48 years old), the study was not well powered to examine individual cancer risks. A Danish study [9] also showed elevated gastric cancer risk after appendectomy, but no altered risks were noted for colon cancer. Conversely, a recent study in Taiwan [25] reported a 14% higher incidence of colorectal cancer among appendectomized patients when compared to matched controls randomly selected from inpatient claims data. The explanation for this discrepancy remains unclear. Besides the geographic differences, possible interpretations for these variations might include different study designs, inadequate statistical precision owing to limited sample size or short follow-up duration, and potential systematic errors, i.e. ascertainment or selection bias. In the current study, compared to the general Swedish population, we only observed a slightly elevated risk (32%) of esophageal adenocarcinoma among appendectomized patients, whereas the risk for esophageal squamous cell carcinoma was noted to be decreased. For all other gastrointestinal cancers, no excess risks could be noted in the study population.

To date, we found no other evidence to support the observed association between appendectomy and subsequent esophageal adenocarcinoma risk. Since in our study there was no obvious tendency of increased cancer risks with longer follow-up duration, it’s very unlikely that the observed linkage could be causative. On the other hand, with the knowledge that obesity could diminish diagnostic accuracy in the diagnosis of acute appendicitis in both children [26] and adults [27], it’s possible that obese individuals experience higher risk of appendectomies during their life time, compared to non-obese individuals. As obesity is a well-established risk factor for esophageal adenocarcinoma, the observed excess risk of esophageal adenocarcinoma among appendectomized patients could reflect the confounding effect of obesity.

The significantly reduced risk of esophageal squamous-cell carcinoma among appendectomized patients was also unexpected. Although we were not able to adjust for those potential confounding factors in the SIR calculation, our additional analysis on main tobacco-related cancer (lung cancer) and main alcohol-related cancer (liver cancer) offered a clue about the differential distribution of these factors in the study population. Despite a few previous studies reported that smoking or passive smoking in children was associated with higher possibility of having acute appendicitis [28], we found no excess risk of lung cancer among appendectomized patients. Instead, a modestly reduced risk of liver cancer was noted, and the reduction was similarly confined to the subgroups among which the reduced risk of esophageal squamous-cell carcinoma was observed. Since we have no data to assess these personal habits among our patients, these results should be interpreted cautiously.

The previously reported association between gastric cancer risk and appendectomy [8, 9], was not replicated in our study. Mechanisms posited by others included confounding by low socioeconomic status, which is also linked to a higher risk of appendectomy [29] and *Helicobacter pylori* (*H*. *pylori*) infection during childhood [30]. Moreover, although no evidence suggests that *H*. *pylori* infection could directly affect the pathogenesis of appendicitis [31], the unspecific abdominal pain caused by *H*. *pylori* [32] might potentially increase the likelihood of having appendectomy. With regard to cancers of the colon or rectum, most prospective studies [9, 33], including ours, found no evidence of an association between appendectomy and colorectal cancer risk, while few others indicated a positive association [25].

To the best of our knowledge, this is the largest population-based study to date on gastrointestinal cancer risk after appendectomy—both in terms of sample size and length of follow-up. The structure of Swedish health care enabled us to include all patients with appendectomy and precise linkages to other complete and high-quality national registers permitted complete follow-up for cancer outcomes as well as for censoring events. However, this study has a few limitations. First, we did not have information on lifestyle factors, and consequently could not adjust for important confounders, such as body weight, smoking/diet habits, and other socioeconomic factors. Given the unexpected altered risks of esophageal adenocarcinoma and esophageal squamous-cell carcinoma, confounding effects of such factors might be substantial. Second, surveillance bias might be another concern. Appendectomy performed due to symptoms or diseases other than appendicitis might *per se* indicate a higher risk of certain cancers. In our analysis, although we have used one-year lag time for ‘wash-out’, such bias might still exist. For instance, the excess risk of cardia cancer which can only be observed in the first few years of follow-up was probably caused by such a surveillance bias.

In conclusion, we observed that, in Sweden, the incidence of appendectomy has decreased during 1987–2009. Also, except for a possible alteration in esophageal cancer risks, no association with stomach, colon, or rectum cancer risks was observed among the appendectomzied patients.

## Supporting Information

S1 Table Sensitivity analysis: Standardized incidence ratio (SIR) and 95% confidence intervals (CIs) for gastrointestinal cancers in the appendectomy cohort, 1987–2009. (DOC)

## Abbreviations

CI: Confidence interval
*H. pylori*: *Helicobacter pylori*
SIR: standardized incidence ratio
