# Supplementary material for: Risk of Gastrointestinal Cancers among Patients with Appendectomy: A Large-Scale Swedish Register-Based Cohort Study during 1970-2009
Source: PLoS One. 2016 Mar 9;11(3):e0151262. doi: 10.1371/journal.pone.0151262 (PMC4784880; doi:10.1371/journal.pone.0151262)
Supplement: S1 Table — (DOC) [file pone.0151262.s001.doc]

Sensitivity analysis: Standardized incidence ratio (SIR) and 95% confidence intervals (CIs) for gastrointestinal cancers in the appendectomy cohort, 1987-2009

|  | 1987-2009 | |
| --- | --- | --- |
|  | Observed cases^*^ | SIR (95% CI) ^§^ |
| **Esophageal cancer, all** | 77 | 1.00 (0.79-1.25) |
| Esophageal adenocarcinoma  Esophageal squamous-cell carcinoma | 41  34 | 1.24 (0.89-1.69)  0.88 (0.61-1.24) |
| **Gastric cancer, all**  Non-cardia gastric cancer  Cardia cancer | 188  143  45 | 0.93 (0.80-1.07)  0.90 (0.76-1.07)  1.05 (0.77-1.41) |
| **Colon cancer, all**  Right-sided colon cancer  Left-sided colon cancer | 709  392  243 | 1.05 (0.97-1.13)  1.11 (1.01-1.23)  0.94 (0.83-1.07) |
| **Rectal cancer** | 360 | 0.97 (0.87-1.07) |

^*^The ﬁrst year of observation and corresponding events were excluded.

^§^ Observed to expected number of cancer cases, based on age- (5-year strata), calendar year- (5-year strata) and sex-specific incidence data in the total Swedish population. Ninety-five percent CIs of SIRs were calculated by assuming that observed cancer occurrence followed a Poisson distribution.
